# Supplementary material for: Enhanced Cd-Accumulation in Typha latifolia by Interaction with Pseudomonas rhodesiae GRC140 under Axenic Hydroponic Conditions
Source: Plants (Basel). 2022 May 29;11(11):1447. doi: 10.3390/plants11111447 (PMC9183143; doi:10.3390/plants11111447)
Supplement: Supplementary file 1 [file plants-11-01447-s001.zip › plants-1687644-supplementary.pdf]

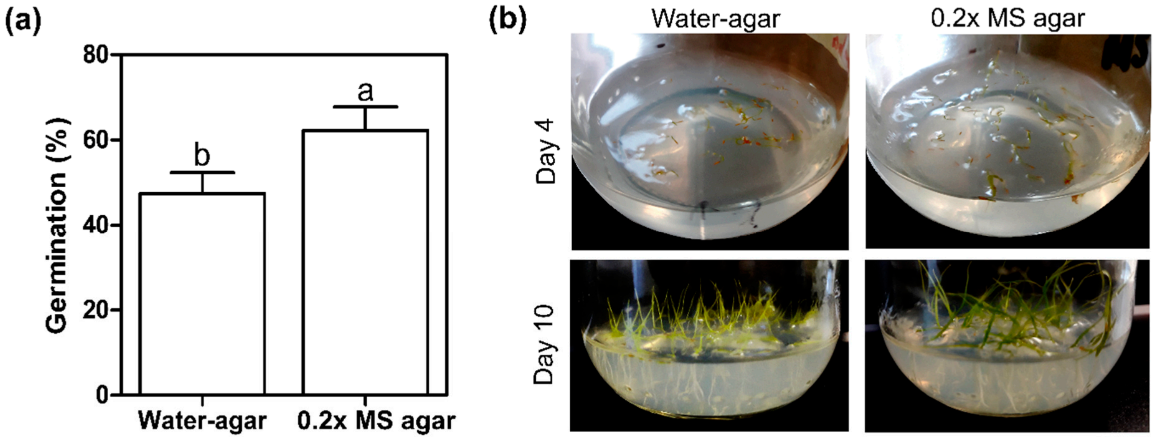

Figure S1. *Typha latifolia* seeds germination. (a) Germination percentage in different culture media after four incubation days, (b) Germinated seeds on water-agar medium and 0.2× MS agar after four and ten incubation days. The values represent the mean ± SD (n = 3). Different letters represent statistically different means ($p < 0.05$, Tukey's test).
